# Clocks do not tick in unison: isolation of *Clock* and *vrille* shed new light on the clockwork model of the sand fly *Lutzomyia longipalpis*

**DOI:** 10.1186/s13071-015-1117-6

**Published:** 2015-10-06

**Authors:** João Silveira Moledo Gesto, Gustavo Bueno da Silva Rivas, Marcio Galvão Pavan, Antonio Carlos Alves Meireles-Filho, Paulo Roberto de Amoretty, Nataly Araújo de Souza, Rafaela Vieira Bruno, Alexandre Afranio Peixoto

**Affiliations:** Laboratório de Biologia Molecular, Instituto Oswaldo Cruz, Fundação Oswaldo Cruz (FIOCRUZ), Rio de Janeiro, Brazil; Mosquitos Vetores: Endossimbiontes e Interação Patógeno Vetor, Centro de Pesquisas René Rachou-Fiocruz, Belo Horizonte, Minas Gerais Brazil; Laboratório de Epidemiologia e Sistemática Molecular, Instituto Oswaldo Cruz, Fundação Oswaldo Cruz (FIOCRUZ), Rio de Janeiro, Brazil; Laboratory of Systems Biology and Genetics, Institute of Bioengineering, School of Life Sciences, École Polytechnique Fédérale de Lausanne, Lausanne, Switzerland; Laboratório de Transmissores de Leishmanioses, Instituto Oswaldo Cruz, Fundação Oswaldo Cruz (FIOCRUZ), Rio de Janeiro, Brazil; Instituto Nacional de Ciência e Tecnologia em Entomologia Molecular (INCT-EM)/CNPq, Rio de Janeiro, Brazil

**Keywords:** *Lutzomyia longipalpis*, Circadian clocks, *Clock*, *vrille*, PAS domain, bZIP domain, bHLH domain

## Abstract

**Background:**

Behavior rhythms of insect vectors directly interfere with the dynamics of pathogen transmission to humans. The sand fly *Lutzomyia longipalpis* is the main vector of visceral leishmaniasis in America and concentrates its activity around dusk. Despite the accumulation of behavioral data, very little is known about the molecular bases of the clock mechanism in this species. This study aims to characterize, within an evolutionary perspective, two important circadian clock genes, *Clock* and *vrille*.

**Findings:**

We have cloned and isolated the coding sequence of *L. longipalpis’* genes *Clock* and *vrille*. The former is structured in eight exons and encodes a protein of 696 amino acids, and the latter comprises three exons and translates to a protein of 469 amino acids. When compared to other insects’ orthologues, *L. longipalpis* CLOCK shows a high degree of conservation in the functional domains bHLH and PAS, but a much shorter glutamine-rich (poly-Q) C-terminal region. As for *L. longipalpis* VRILLE, a high degree of conservation was found in the bZIP domain. To support these observations and provide an elegant view of the evolution of both genes in insects, phylogenetic analyses based on maximum-likelihood and Bayesian inferences were performed, corroborating the previously known insect systematics.

**Conclusions:**

The isolation and phylogenetic analyses of *Clock* and *vrille* orthologues in *L. longipalpis* bring novel and important data to characterize this species’ circadian clock. Interestingly, the poly-Q shortening observed in CLOCK suggests that its transcription activity might be impaired and we speculate if this effect could be compensated by other clock factors such as CYCLE.

**Electronic supplementary material:**

The online version of this article (doi:10.1186/s13071-015-1117-6) contains supplementary material, which is available to authorized users.

## Findings

Understanding the dynamics of pathogen transmission to the human hosts is a crucial step towards the development of effective tools to control vector-borne diseases. From this perspective, the study of vector behavior, as well as its genetics underpinnings, is key for any vector control program. One of the most relevant behavior traits across insect taxa is the circadian rhythmicity, which is depicted by well-defined daily patterns of locomotion, flight, adult emergence, olfactory response and other biological phenomena [1]. In tropical disease vectors, such as mosquitoes and sand flies, feeding habits and hematophagic behavior also follow a precise circadian schedule, often resulting in the outline of species-specific temporal niches [2–6].

For the last couple of decades, great effort has been made to unravel the molecular and cellular bases of the endogenous clock behind the circadian rhythms. In insects, the most comprehensive information regarding the molecular clock comes from the research with *Drosophila melanogaster*. Due to state-of-the-art genetic tools developed for this species, the core clock factors could be identified and fit in a pacemaker model consisting of self-sustained feedback loops that act on the regulation of gene expression [7, 8]. Briefly, the transcription factors *Clock* (*Clk*) and *cycle* (*cyc*) interact at the pacemaker’s core and activate the expression of several clock genes, such as *period* (*per*), *timeless* (*tim*), *vrille* (*vri*), *Par domain protein 1* (*Pdp1*) and *clockwork orange* (*cwo*). Along the day, the proteins encoded by these genes feedback to modulate CLK/CYC transcription activity, thereby generating a molecular oscillation in their own expression. This model acquires extra layers of complexity with post-transcriptional and post-translational regulation, as well as cellular communication within neuronal clusters, providing a daily harmonious modulation of rhythmic behavior [7, 8].

Despite the good amount of data on the behavioral rhythms of tropical disease vectors [2–6], few studies have focused on the cellular and molecular mechanisms underlying them. The sand fly *Lutzomyia longipalpis* (Diptera, Psychodidae, Phlebotominae), main vector of visceral leishmaniasis in the New World, was the first insect vector to have clock components identified at the molecular level, with full characterization of the *cyc* orthologue [9, 10] Interestingly, gene expression assessment of these components revealed aspects of the pacemaker that are different from the *Drosophila* model: 1) *Clk* mRNA peaks at an opposite phase in the two species; and 2) *cycle* expression exhibits a significant circadian oscillation in sand flies, whereas in *Drosophila* it is constitutive [9, 10]. Nevertheless, these are probably not the only differences driving the distinct activity pattern between the two species, with *D. melanogaster* being mainly diurnal and *L. longipalpis* predominantly active at dusk/night [9]. The identification and functional characterization of other clock genes is key for the construction of a complete profile of the molecular pacemaker regulating the behavior in sand flies.

In the current work we report the isolation of the coding sequences of *Clk* and *vri* orthologues in *L. longipalpis* and their phylogenetic placement in Insecta (Neoptera infraclass) trees. Sand fly specimens were derived from a natural population of Lapinha Cave (longitude 43°57′W, latitude 19°03′S; approximate altitude 700 m), a non-endemic area located in Sumidouro State Park, Minas Gerais, Brazil. Sand flies from this location were used as reference because they have been extensively studied and can be collected in greater abundance than other populations from endemic areas. Nucleotide sequences were amplified by PCR using both degenerate and specific primers (Additional file 1), following a “gene walking approach” according to Gentile et al. [11]. The 3′ region was tackled by using the oligo-dT primer and the 5′ region by means of the “5′ Race System for Rapid Amplification of cDNA Ends” kit (Life Technologies), although the latter did not work well for *Clk*. In this case, the 5′ region was obtained by PCR with the degenerate primer 5CLKdeg13, which anneals to the beginning of the coding sequence. Fragment sequences were subjected to fluorescent dye-terminator cycle sequencing reactions (ABI Prism® BigDye® Terminator v3.1 Cycle Sequencing Kit, Applied Biosystems), and run on an ABI 3730 automated sequencer. Nucleotide sequences were compared to the *D. melanogaster* database (www.flybase.org/blast), confirming homology to *Clk* and *vri*. Subsequently, these sequences were trimmed for poor quality fragments and plasmid sequences, and then assembled in unique contigs using the Staden software [12].

We have obtained the entire nucleotide coding sequence of *L. longipalpis Clk* and *vri* [GenBank: KR706373; KR706372]. *L. longipalpis Clk* encodes a putative protein sequence of 696 amino acids, enclosing the expected bHLH (basic Helix-Loop-Helix) (residues 12–63) and PAS (*per-arnt-sim*) (residues 84–351) domains, the former involved in DNA binding and the latter in protein dimerization [13]. The glutamine-rich (poly-Q) region, which is believed to be involved in CLK transcription activation [13], is much shorter or even absent in this species. With respect to *L. longipalpis’ vri*, the predicted protein sequence contains 469 amino acids and carries the characteristic bZIP (basic Leucine Zipper) domain (residues 112–168), which mediates DNA binding [14]. All these functional domains were confirmed using the Pfam v27.0 database [15].

**Fig. 1:**
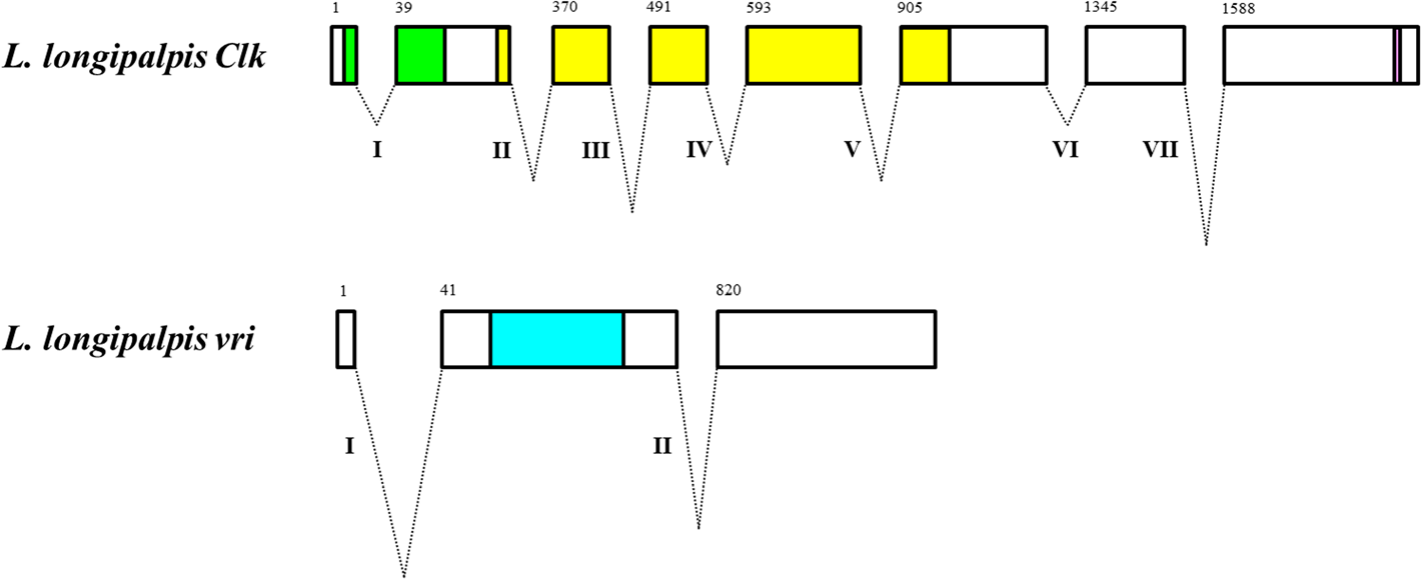
Schematic representation of *L. longipalpis Clk* and *vri* genes. The coding region of *L. longipalpis Clk* gene includes 8 exons and 7 introns, while *L. longipalpis vri* contains 3 exons and 2 introns. The numbers above the exons represent the position where they start, based on the coding sequence. Introns are presented by Roman numerals. *Clk* intron sizes are as follows: I (62 bp), II (398 bp), III (497 bp), IV (283 bp), V (357 bp), VI (72 bp), VII (538 bp). For *vri*, intron sizes are: I (11139 bp) and II (969 bp). Functional domains are colored in green (bHLH), yellow (PAS) and purple (poly-Q) for *Clk*, and in blue (bZIP) for *vri*

Soon after the experimental isolation of *Clk* and *vri* was achieved, the genome project of *L. longipalpis* released its first assembly (LlonJ1), including a number of draft contigs and scaffolds (https://www.vectorbase.org/organisms/lutzomyia-longipalpis/jacobina/llonj1). Using our sequences as queries in BLAST searches, we were able to locate *Clk* and *vri* in the LlonJ1.1 gene set. Due to misassembly issues, *Clk* sequence maps to two predicted genes: LLOTMP006614 (Scaffold48: 204225–210403 forward strand) and LLOTMP000502 (Scaffold108: 104,185–106,138 forward strand), both incorrectly annotated. On the other hand, this was not an issue with *vri*, which is annotated as LLOTMP007416 (Scaffold568: 62113–76422 forward strand). The comparison of our sequences to the currently available genomic DNA allowed the identification of exons, introns and their boundaries. Hence, while *Clk* is structured in 8 coding exons and 7 introns, *vri* contains 3 coding exons and 2 introns (Fig. 1).

In order to evaluate the degree of CLK and VRI conservation in insects, giving emphasis to vector species, the predicted protein sequences of *L. longipalpis* were aligned to their orthologous counterparts in eight neopteran insect species (Additional file 2), using the Multalin algorithm [16]. The Blosum62 substitution model [17], with default parameters of gap penalties imposed, was used to construct alignment matrices. No penalty was charged for terminal gaps.

The multiple sequence alignment (MSA) of CLK orthologues (Additional file 3) revealed highly conserved regions related to bHLH and PAS functional domains, reaching an identity degree of 63 and 39 %, respectively, among the species analyzed. The C-Terminal region, on the other hand, is quite divergent, with low amino acid conservation. Equally variable is the size and structure of the poly-Q sequence found in this region, which is abundant in some species (e.g. *D. melanogaster*) but seems to be reduced or absent in *L. longipalpis*. The MSA of VRI orthologues (Additional file 4) also revealed regions with a high-degree of conservation, reaching 77 % of identity in the bZIP domain.

Consensus phylograms were constructed for *Clk* and *vri* orthologous genes, using a Bayesian inference with the BEAST v1.8 package [18] and the Maximum-likelihood (ML) approach designed in PHYML v3.0 [19]. All analyses were run in the Cipres Science Gateway environment [20]. Briefly, a prior tree was randomly generated in both types of phylogenetic reconstructions. The Yule process of speciation was imposed for Bayesian trees. In this case, three independent runs were performed for 4×10^7^ generations, with a burn-in of 10 %. Proper mixing of chains and convergence of parameters were confirmed by calculating the effective sample size (ESS) in Tracer v1.6 [21]. All considered parameters had ESS >2×10^4^. Maximum credibility tree was constructed with 12,000 trees (burn-in = 1,200). Statistical support for clades was assessed by the posterior probability method and 1,000 bootstrap replicates in Bayesian and ML trees, respectively. ProtTest v3.0 [22] was used to elect the joint transform-domain-translated with unequal frequencies and four-gamma parameters (JTT + Γ + I) was the best-fit model of amino acid substitution (Akaike Information Criterion corrected for number of samples and number of amino acids of each sequence) for *Clk* and *vri* phylogenetic reconstructions. The Poisson model available in MEGA 5 [23] was used to estimate a species distance matrix and a heatmap was constructed in the R environment [24].

**Fig. 2:**
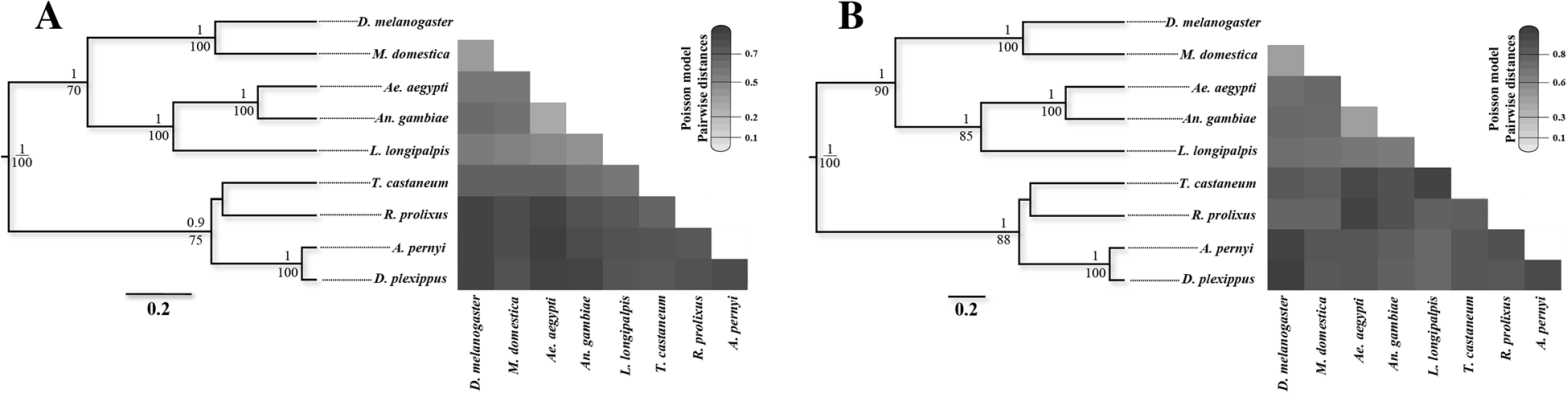
Consensus phylograms based on the Bayesian inference Maximum-likelihood (ML) approach for *Clk* (**a**) and *vri* (**b**) genes and heatmaps illustrating pairwise genetic distance values. Posterior probabilities >0.9 and bootstrap values >70 are shown for each node (above and below each node, respectively). Darker colors of squares in the heatmaps represent larger distances (see scale bar)

Phylogenetic analyses corroborated the previously known neopteran insect systematics (Fig. 2). The Nematocera suborder was recovered in a well-supported cluster (PP = 1; bootstrap = 85/100) containing *Clk* and *vri* orthologues of *L. longipalpis* and of *Ae. aegypti* and *An. gambiae* (overall pairwise distance = 30 % and 57 % for *Clk* and *vri*, respectively). These sequences were closer to each other than to the other dipterans *D. melanogaster* and *M. domestica* (infraorder Muscomorpha; overall distance = 53–57 % and 74–77 % for *Clk* and *vri*, respectively). All dipteran sequences were well separated from others belonging to Coleoptera and Hemiptera (*T. castaneum* and *R. prolixus*, respectively, which clustered together), and to Lepidoptera orders (*A. pernyi* and *D. plexippus*), with distance values above 50 % for *Clk* and 80 % for *vri* ortolog sequences.

We believe that the identification, annotation and phylogenetic analyses of *Clk* and *vri* orthologues in *L. longipalpis* contribute with novel and important data towards a better understanding of this species’ biology and its molecular clockwork. The coding sequences of the two clock genes provide useful information for probe design and open new possibilities for circadian expression and functional assays, as well as population genetic studies. Clock genes are suitable markers for identifying sibling species, since they are involved in species-specific behaviors that may lead to reproductive isolation and ultimately result in speciation [25–27].

A list of future prospects includes the silencing of *Clk* and *vri* expression (through RNAi) and analysis of the physiological and behavioral output of this manipulation. Also, the identification of conserved regions across the genes and the correct assignment of exon-exon boundaries are valuable information to design Exon-Priming Intron Crossing (EPICs) for molecular systematics studies in sand flies.

Finally, our findings also revealed a great divergence in CLK C-terminal sequence, with variation in the poly-Q position and length. In order to further investigate the functional significance of this region in sand flies, where it is much reduced in size, CLK chimeras can be constructed and tested in cell reporter assays. This will allow proper evaluation of its capacity to bind promoter elements and activate gene expression. Similarly, CLK chimeras in transgenic *Drosophila* lines may help to assess the ability of rescuing *Clk*-null mutant phenotypes. Curiously, a transcriptional activation domain very common in vertebrates (a.k.a. BCTR) is found in *L. longipalpis cyc* [10]. Assuming that CLK and CYC dimerize like in *Drosophila,* the BCTR could function to compensate the absence of CLK’s poly-Q and provide an alternative model for clock genes regulation in the sand fly clockwork.

## Additional files

Additional file 1:
**Degenerate and specific primers used to amplify** ***Clk*** **and** ***vri*** **homologous fragments in** ***L. longipalpis.*** (DOCX 15 kb) Additional file 2:
***Clk*** **and** ***vri*** **orthologues in neopteran insect species.** (DOCX 28 kb) Additional file 3:
**Multiple sequence alignment of CLK orthologues in** ***L. longipalpis*** **[GenBank: KR706373],** ***Ae. aegypti*** **[GenBank:XP_001662706],** ***An. gambiae*** **[GenBank:XP_315720],** ***D. melanogaster*** **[GenBank:AAF50516],** ***M. domestica*** **[GenBank:XP_005180856],** ***R. prolixus*** **[VectorBase:RPRC002110],** ***D. plexippus*** **[GenBank:EHJ69324],** ***A. pernyi*** **[GenBank:AAR14936]** **and** ***T. castaneum*** **[GenBank:NP_001106937].** The Highlighted regions correspond to the functional domains bHLH (green), PAS (yellow) and poly-Q (purple). Note that PAS spans subunits A and B, both highly conserved in insects. The poly-Q, on the contrary, is very diversed in position and length. Text colors correlate to conservation thresholds for a position. In red font, highly conserved residues (threshold=90 %). In blue font, weakly conserved residues (threshold=50 %). (PDF 195 kb) Additional file 4:
**Additional file 2.** Multiple sequence alignment of VRI orthologues in *L. longipalpis* [GenBank: KR706372], *Ae. aegypti* [GenBank:XP_001661622], *An. gambiae* [GenBank:XP_317705], *D. melanogaster* [GenBank:NP_477191], *M. domestica* [GenBank:XP_005180212], *R. prolixus* [VectorBase:RPRC000393], *D. plexippus* [GenBank:AAT86041], *A. pernyi* [GenBank:AAS92609] and *T. castaneum* [GenBank:EFA11543]. The Highlighted region (blue) corresponds to bZIP functional domain. Text colors correlate to conservation thresholds for a position. In red font, highly conserved residues (threshold=90 %). In blue font, weakly conserved residues (threshold=50 %). (PDF 171 kb)
