# Supplementary material for: Clocks do not tick in unison: isolation of Clock and vrille shed new light on the clockwork model of the sand fly Lutzomyia longipalpis
Source: Parasit Vectors. 2015 Oct 6;8:505. doi: 10.1186/s13071-015-1117-6 (PMC4595053; doi:10.1186/s13071-015-1117-6)
Supplement: Additional file 1: — Degenerate and specific primers used to amplify Clk and vri homologous fragments in L. longipalpis. (DOCX 15 kb) [file 13071_2015_1117_MOESM1_ESM.docx]

**Table 1**

Degenerate and specific primers used to amplify *Clk* and *vri* homologous fragments in *L. longipalpis.*

| ***Clk*** |  |
| --- | --- |
| **Degenerate primers** | **Sequence (5’ 🡪 3’)** |
| 5CLKdeg1 | AARATGGAYAARWSNACNGT |
| 5CLKdeg3 | SNGGNTAYGAYTAYTAYCA |
| 3CLKdeg4 | TGYTGYTGYTGCATRTG |
| 3CLKdeg10 | TCNGTYTGNARCCADATCCA |
| 3CLKdeg12 | ARRAANGCNGGYTTCCARTC |
| 5CLKdeg13 | ATGGAYGARGAYCCNGAYGA |
| 5CLKdeg14 | GAYGAYAARGAYGAYACNAA |
| **Specific primers** | **Sequence (5’ 🡪 3’)** |
| 3GSPCLK3 | CGTGATCTCAGTGCAATCTCG |
| 5LLCLK1 | CTAACAAAAGGGCAGCAG |
| 5LLCLK2 | GCGAAGGAACATCCTGCTAC |
| 3LLCLK3 | CCTTCGCCTTTTTGCATC |
| 5LLCLK4 | AACTCCTCATGGCTCGCTAC |
| 3LLCLK4 | AAACCCTCCGTAGCTCTTC |
| 3LLCLK5 | CGTTCACAATCGACGGAAG |
| 5LLCLK6 | CGAGATTGCACTGAGATCAC |
| 5LLCLK7 | ACTGAGATCACGAAGCCAC |
| 5LLCLK10 | AGCAACATTCCGATGCTGAG |
| 5LLCLK12 | CTTCTCCTGCCACCTGAAAC |
| 3LLCLK12 | TGTCCCAAGGACTTCAAAGG |
| 3LLCLK14 | AGCCAGCAAATTGAACAAGC |
| oligo dT-EcoRI | TTTTGAATTCTTTTTTTTTTTTTTTTTTTT |
|  |  |
| ***vri*** | |
| **Degenerate primers** | **Sequence (5’ 🡪 3’)** |
| 5Vrideg01 | AARGAYGANWSNTAYTGGGA |
| 5Vrideg02 | AAYGAYATGGTNYTNGARCA |
| 3Vrideg03 | ATNGCRTCNARYTGNGCYTT |
| 3Vrideg04 | GCNGCRTCYTTRTCNCC |
| 3Vrideg05 | CCRTCRTYCCANGCNGG |
| 3Vrideg06 | TTDATNGKNGCNACY |
| **Specific primers** | **Sequence (5’ 🡪 3’)** |
| antivri5a | GGGATAGACGAAGACGCAAC |
| antivri5b | GACGCAACAATGAAGCAGCC |
| antivri3a | GACGGGGACTTTGATGCAGT |
| antivri3b | CAGTGTTCTCAGTAGACCCA |
| 3vrirace1 | GGACGCCATAAAGGA |
| 3vrirace2 | TGGAGCAGCGAGTTGTGGAG |
| 3vrirace3 | AAGCAGCCAAGAGATCCCGT |
| 5llvri01 | TAAACCTCTCACGTCGTC |
| 5llvri02 | ACAAGAGTCATCTGGGTG |
