# Supplementary material for: Clocks do not tick in unison: isolation of Clock and vrille shed new light on the clockwork model of the sand fly Lutzomyia longipalpis
Source: Parasit Vectors. 2015 Oct 6;8:505. doi: 10.1186/s13071-015-1117-6 (PMC4595053; doi:10.1186/s13071-015-1117-6)
Supplement: Additional file 2: — Clk and vri orthologues in neopteran insect species. (DOCX 28 kb) [file 13071_2015_1117_MOESM2_ESM.docx]

**Table 2:** *Clk* and *vri* orthologues in neopteran insect species.

| **Gene** | **Species** | **Nucleotide Accession # ^1^** | **Protein Accession # ^1^** | **mRNA length (base pairs)** | **Exon count ^2^** | **GC content (%) ^3^** | **Protein length (amino acids)** |
| --- | --- | --- | --- | --- | --- | --- | --- |
|  |  |  |  |  |  |  |  |
|  | *Aedes aegypti* | XM_001662656 | XP_001662706 | 2793 | 6 | 50 | 900 |
|  | *Anopheles gambiae* | XM_315720 | XP_315720 | 3860 | 6 | 58 | 1030 |
|  | *Drosophila melanogaster* | NM_079240 | AAF50516, NP_523964 | 4422 | 7 | 47 | 1023 |
| ***Clk*** | *Musca domestica* | XM_005180799 | XP_005180856 | 3722 | 7 | 43 | 1081 |
|  | *Rhodnius prolixus* | RPRC002110-RA | RPRC002110-PA | 1749 | 16 | 37 | 582 |
|  | *Danaus plexippus* | AGBW01007006 | EHJ69324 | 1809 | 13 | 49 | 602 |
|  | *Antheraea pernyi* | AY330486 | AAR14936 | 2157 | N/A | 46 | 611 |
|  | *Tribolium castaneum* | NM_001113466 | NP_001106937 | 1746 | 9 | 47 | 581 |
|  |  |  |  |  |  |  |  |
|  |  |  |  |  |  |  |  |
|  | *Aedes aegypti* | XM_001661572 | XP_001661622 | 2087 | 2 | 49 | 585 |
|  | *Anopheles gambiae* | XM_317705 | XP_317705 | 1932 | 2 | 65 | 608 |
|  | *Drosophila melanogaster* | NM_057843 | NP_477191 | 3841 | 3 | 50 | 729 |
| ***vri*** | *Musca domestica* | XM_005180155 | XP_005180212 | 3548 | 3 | 45 | 796 |
|  | *Rhodinius prolixus* | RPRC000393-RA | RPRC000393-PA | 1203 | 2 | 43 | 400 |
|  | *Danaus plexippus* | AY576272 | AAT86041 | 1095 | 2 | 55 | 364 |
|  | *Antheraea pernyi* | AY526608 | AAS92609 | 1710 | N/A | 51 | 364 |
|  | *Tribolium castaneum* | CM000276 | EFA11543 | 1315 | 2 | 57 | 338 |

1. Accession numbers refer to GenBank^®^ (http://www.ncbi.nlm.nih.gov/genbank/), except for the orthologues in *Rhodnius* *prolixus* which refer to VectorBase (https://www.vectorbase.org/).
2. Calculated according to the coding region.
3. Calculated according to the sequences provided by the Nucleotide Accession #.

N/A, not available.
