# Supplementary material for: Clocks do not tick in unison: isolation of Clock and vrille shed new light on the clockwork model of the sand fly Lutzomyia longipalpis
Source: Parasit Vectors. 2015 Oct 6;8:505. doi: 10.1186/s13071-015-1117-6 (PMC4595053; doi:10.1186/s13071-015-1117-6)
Supplement: Additional file 3: — Multiple sequence alignment of CLK orthologues in L. longipalpis [GenBank: KR706373], Ae. aegypti [GenBank:XP_001662706], An. gambiae [GenBank:XP_315720], D. melanogaster [GenBank:AAF50516], M. domestica [GenBank:XP_005180856], R. prolixus [VectorBase:RPRC002110], D. plexippus [GenBank:EHJ69324], A. pernyi [GenBank:AAR14936] and T. castaneum [GenBank:NP_001106937]. The Highlighted regions correspond to the functional domains bHLH (green), PAS (yellow) and poly-Q (purple). Note that PAS spans subunits A and B, both highly conserved in insects. The poly-Q, on the contrary, is very diversed in position and length. Text colors correlate to conservation thresholds for a position. In red font, highly conserved residues (threshold=90 %). In blue font, weakly conserved residues (threshold=50 %). (PDF 195 kb) [file 13071_2015_1117_MOESM3_ESM.pdf]

```
                                   basic                         HLH
L.longipalpis   MDEDPDDKDDSKR------KSRNLSEKKRRDQFNLLVNELSSMVSSNNRKMDKSTVLKSTIAFLKNHNEIALRSRSHEIQEDWKPSFLSNEEFTHLILEA
Ae.aegypti      MEDDPDDKDDTKRFVVYDSKSRNLSEKKRRDQFNLLVNELSSMVSSNSRKMDKSTVLKSTIAFLKSHNEIAVRSRVHEIQTDWKPSFLSNEEFTHLILEA
An.gambiae      MDEDPDDKDDTKR------KSRNLSEKKRRDQFNLLVNELSSMVSSNSRKMDKSTVLKSTIAFLKSHNEIAVRSRVHEIQTDWKPSFLSNEEFTHLILEA
D.melanogaster  MDDESDDKDDTKR------KSRNLSEKKRRDQFNSLVNDLSALISTSSRKMDKSTVLKSTIAFLKNHNEATDRSKVFEIQQDWKPAFLSNDEYTHLMLES
M.domestica                                                       MDKSTVLKSTITFLKHHNEATDRSKVFEIQQEWKPSFLSNDEFTQLMLES
A.pernyi        MDDDGDEKDDSKR------RTRNLSEKKRRDQFNLLINELSSLVTSNSRKMDKSTVLKSTISFLRNHNEITVRSRAHDIQDDWKPTFLSNEEFTYLVLEA
D.plexippus     MDDDGDDKDDTKR------RTRNLSEKKRRDQFNMLVNELGSMVSTNNRKMDKSTVLKSTISFLKNHNEITVRSRAHDVQEDWKPAFLSNEEFTYLVLEA
R.prolixus      MDEDTDDKDDSKR------KTRNLSEKKRRDQFNLLINELGSMVSTNNRKMDKSTVLKSAISYLKNHNDVAVLSRANEIQEDWKPSFLSNEEFIHLFLEA
T.castaneum     MDDDSDDKD-SKR------KSRNLSEKKRRDQFNLLVNELSSMVATGSRKMDKSTVLKSTIAFLKNHNEIAVRSRVNEIQEDWKPTFLTNEEFTHLILEA

                         PAS A
L.longipalpis   LDGFIMVFSSTGRIFYASESITSLLGHLPSDLLNMTIYDLAYEEDHSELYNVLLNPAAITDPLQNNLTRENQILFSCHLKRGSSENDNRDGLTYELVQFV
Ae.aegypti      LDGFIIVFSSTGRVFYASESITSLLGHLPSDLLNMTVYDMVYEDDQNDLYNILLNPAAVVDPLQTGISRENQVTFSCYIKRGTADY--RTEVSYELVQFT
An.gambiae      LDGFIIVFSSTGRVFYASESITSLLGHLPSDLLNMTVYDMVYEDDQNDLYNILLNPTTVVDPLQTGISRENQVTFSCYIKRGTVDY--RADVSYEHVQFT
D.melanogaster  LDGFMMVFSSMGSIFYASESITSQLGYLPQDLYNMTIYDLAYEMDHEALLNIFMNPTPVIEPRQTDISSSNQITFYTHLRRGGMEK--VDANAYELVKFV
M.domestica     LDGFIIVFGSSGSIFYTSDSVTAQLGYLPCDLMKMTIFDLSYEMDHEILLNMFLNPKPVIAPMQTDIGARNQITFFLHLKRRGTED--GDRNSFELVKFV
A.pernyi        LEGFVVVFSTSGRIHYVSESISSLLGYNPVDIINKSLFELVFEEDQQTLYSLLQSPGNITDP--THTGKENEIQFQCHIRRGGSSE-YGEDVAYELIQFN
D.plexippus     LEGFVMVFSASGCIYYVSESVTSLLGHTPGDIINKSIFDLAFVDDRPNLYNILQNGGTLDPT--QVVTTDNPISFRCRLQRG-TLD-FRDEVTYELVQFD
R.prolixus      LDGFIIVFSSTGRILYVSESVTSLLGYFPCDMLAMTLYDLVYEGDHSELYNML-----LNPS--TNFETDNELSFMCHCKRG-GID-IKEEPVYEIILFI
T.castaneum     VDGFIMVFSASGQIFYASESITSLLGHLPNQVLNMTIYEMANEEEHSHLYNIL-----LTPS-----EDQGQVSFSCHLRRG-DPD-SKQNPSFELVHFV

                                                                                                    PAS B
L.longipalpis   GYFRT---------------DVDIENMLPNARNCIYPSDADTRLIFVGTGRLQTPQLIREMSIVDSTKSEFTSRHSLEWKFLFLDHRAPPIIGYLPFEVL
Ae.aegypti      GYFSEYDRWSSCGS------DVDADSLMTTSRFSGYMTDADTRLIFVGTGRLQTPQLIREMSIVDSSKSEFTSRHSLEWKFLFLDHRAPPIIGYLPFEVL
An.gambiae      GYFR---------S------DVDTESLMTTSRFSGYTSDADSRLVFVGTGRLQTPQLIREMSIVDNTKSEFTSRHSLEWKFLFLDHRAPPIIGYLPFEVL
D.melanogaster  GYFRNDTNTSTGSSSEVSNGSNGQPAVLPRIFQQNPNAEVDKKLVFVGTGRVQNPQLIREMSIIDPTSNEFTSKHSMEWKFLFLDHRAPPIIGYMPFEVL
M.domestica     GYFRNDAKIDVVAQ----NTQTSLPRALQR--NSASAIDLDHKLIFVGTGRLQMPQMIREITVTDPTCSEFTSKHSMEWKFLFLDQRAPPIIGYMPFEVL
A.pernyi        GHFRSNVE------------SLH------ADDLSHYRQGSDNRLLFVCTGRLSNPQLIRDVSLVDSSRNEFTSRHSLEWKFLFLDRRAPPIIGYLPFEVL
D.plexippus     GHFRKNLE------------S-N------ENGHHSYQDEHESRLLFVCTGRLYMPQLVRDVSLVDTIRSEFTSRHSLEWKFLFLDHRAPPIIGYLPFEVL
R.prolixus      GHFGSDVD------------SLHLNNGVSSNRYSTYSAEAENRWVFLGTGRLQIPQLITELSVIDSSKCEFTSRHSLEWKFLFLDHRAPPIIGYLPFEVL
T.castaneum     GYFRSDED------------MVQ-----SENRYSGYSGEADTRLVFVGTGKIKTPRLIREMPLVDSSKSEFTSRHSLEWKFLFLDHRAPPIIGYLPFELL

L.longipalpis   GTSGYDYYHFDDLEKVITCHEALMQKGEGTSCYYRFLTKGQQWIWLQTRFYITYHQWNSKPEFVVCTHRVVSYADVMKQSRN-----GGDAEMTDSLEKD
Ae.aegypti      GTSGYDYYHFDDLEKVVSCHEALMQKGEGTSCFYRFLTKGQQWIWLQTRFYITYHQWNSKPEFVVCTHRVVSYADVMKQMRNQAGGEGKFSEDTDSVSI-
An.gambiae      GTSGYDYYHFDDLEKVVACHEALMQKGEGTSCYYRFLTKGQQWIWLQTRFYITYHQWNSKPEFVVCTHRVVSYADVMKQMRNQTAGDSKFSEDADSISVH
D.melanogaster  GTSGYDYYHFDDLDSIVACHEELRQTGEGKSCYYRFLTKGQQWIWLQTDYYVSYHQFNSKPDYVVCTHKVVSYAEVLKDSRKEGQKSGNSNSITNNGSSK
M.domestica     GTSGYDYYHFDDIDDIVSCHEKLMQEGKVKSGYYRFLTKGHQWIWLQTDSYISYNQYTTKPEYVVCTHKVINYIDVLKYGTYDRNNPASSAKPSSSSSSS
A.pernyi        GTSGYDYYHFDDLEKVITCHEALMQKGELTSCYYRFLTKGQQWIWLQTRFYITYHQWNSKPEFIVCTHRVVSYTDMEKDMKQESVEGEV-MSDADLNRGT
D.plexippus     GTSGYDYYHFDDLEKVVSCHEALMQKGELTSCYYRFLTKGQQWIWLQTRFYITYHQWNSKPEFVVCTHRVVSYADIIKSTKQERTETEESVRDCDHNGSS
R.prolixus      GTSGYDYYHVDDLDKVVNCHETVMQKGECTSCYYRFLTKGQQWIWLQTRFYITYHQWNSKPEFIVCSHRVVSYVNVLKQT----------LENTDYNNIF
T.castaneum     GTSGYDYYHVDDLDNIIGHKALMQKGEGTSCFYRFLTKGQQWIWLQTRYYITYHQWNSKPEFIVCTHRVVSYVDVMKQ------------------NRK

L.longipalpis   LEEVDGTTDRQMPTTANSPWSSKSSRMSRFAVTPGSPSV----KSRQRYNTYHGTGSDS--SVSAESHTSRHSLITQYSSRSRLRQHSHPSKTTQ-----
Ae.aegypti      GVERKFQPSSSQSLLATSPWSSKSSRTSRIAPTPGGSPTGAPSRGRHRYNTYQGPGSDSATSMSAESHVSRQSMMTQHS-SVIRSNTFHSKPSSQDGSS-
An.gambiae      GVERKFQQSSSQSLLATSPWSSKSSRTSRIAPTPGVSPTGLSNRGRHRYNTYHGPGSDSATSISTESHTSRQSLVTQHSRSRMRTSTFPSKVSSHGSQSD
D.melanogaster  VIASTGTSSKSAS-----------------------ATTTLRDFELSSQNLDSTLLGNSLASLGTETAA------TSPAVDSSPMWSASAVQPSG--SCQ
M.domestica     ALAPREMANKNPTNTIDTTLPVTPTDINADTVPTLTASTAAVACTTPNNNMITVSSSNNIYSHSTNLIGDILRNETSPSLDSSTLWTNPPTPTGAGQSCQ
A.pernyi        IEDATAENPVVSPSPPYVSEGSD---------------------PYINSYQPLSQPASVKSTSAS-STTGTASTVQTVSTTA-VSWSRASQPPYA-----
D.plexippus     LKDPSTEDAMVPVSPSYMSEASD---------------------AFATSYNSMSKLASVKSAATSGSTSATVATLGTAITTASATWPPRSSYLLY-----
R.prolixus      DEPEKVKSYPTRTSP--WSSKSS---------------------GNFTVGKREKQGGSDSNSMSAESPLSRHSQLTGVSAHSTRGGQAGSGSLVK-----
T.castaneum     EETGVTTSGTSPGTP--WPKNTK---------------------SNIRQGT-----ASECTSMSADSPTSRQSVMTQFSVKSESNQPKPPSQMLQ-----

L.longipalpis   -----------------------------------------------------------------------------------------------SQGMH
Ae.aegypti      QHTAAHFILQQ----------------------------------------------------------------QMMPNPQQPSPESFQQMLATTQHQI
An.gambiae      RNSMSHFLLQQPQQSHGGHQLPSGQLQHQQQHLPQQQQHQQQQQQQPQHPLQHQQPHPLLQPHQQLQGHQAQQPHQQLPPQQQPHPAMIQNPPDVYPHQL
D.melanogaster  INPLKT-SRPASSYGNISSTGISPKAKRKCYFYNNRGNDSDSTSMSTDSVTSRQSMMTHVSSQSQRQRS-----------HHREHH----RENHHNQSHH
M.domestica     LQAIKSNSRPASSYGNISSTGVSPNVKRKRYMYNCRGNESDSTSMSAESGTSRQSLMTHMSTGRQRFTSNLGHSNYSSSGHHQQQNLTSTNPHHHSHFSH
A.pernyi        -----------------------------------------------------------------------------------------------PSVSD
D.plexippus     -----------------------------------------------------------------------------------------------TTGSD
R.prolixus      -----------------------------------------------------------------------------------------------NESKG
T.castaneum     -----------------------------------------------------------------------------------------------PQQPQ

L.longipalpis   QMTQASGQTASQNVHQRQ--------------------------------------------------------VQAP--QMMGPT-FLEPPQYITAIP
Ae.aegypti      RP-------------------------------------------------------------------PPAAAIITPPVTQIISPAGFIEPQQYLAAIP
An.gambiae      QTQHPLQQQQQQQQQSQQPSQQQQSHQQLAGLQQPGTVQQQQSHQQPQHQQPQQQQPPLPQPSTTAALRPPPATIITPPVTQILTATGFIEPQQYLTAIP
D.melanogaster  HMQQQQQHQNQQQQHQQHQQLQQQLQHTVGTPKMVPLLP-----IAST-----------QIMAGNACQFPQPAYPLASP--QLVAPT-FLEPPQYLTAIP
M.domestica     HQQNHSQQQSSQHAQSLHQQQQIQMNSSPQGQRMFQSISQHPAIVQGS-----------PIGGTGTCQFSQPTFPIRSP--QIVAPP-FIESPQYLTAIP
A.pernyi        TASVSGESRSSARNNSHD-----------------------------------------------------------------------TRKVSEPVLVP
D.plexippus     TTSVSGGSRSSQRNSSQE-----------------------------------------------------------------------LQRLPEPALVP
R.prolixus      NKPVERNDGYHPHSFA----------------------------------------------------------------------------V---VGVP
T.castaneum     PLQPQNQIQPSNHQFLEP-----------------------------------------------------------------------PQYV---AAIP

L.longipalpis   VQPVLAPAPFTAAATVIQPLQ-------------PTELIHSGVVMTSAQSQIQDQLQRKHEELQHLIVQQQEELRRVSEQLLMARYGLLPSIVNVTLAPS
Ae.aegypti      VQPVAGFPPDSSTG-VLSPIPPTSP-AAYAV-HPTVPSAAGSVVLTPAQNQVQDQLQRKHEELQHLIMHQQEELRRVQEQLLMARYGLLPSIVTLPFTSA
An.gambiae      VQSVAGFT-ETAPG-VLSPIQSTSPHGAYAVAHHPAVTGTGGVVLTPSQNQVQDQLQRKHEELQHLILQQQEELRRVQEQLLMARYGLLPSIVSLPFPPG
D.melanogaster  MQPV--IAPFPVAP-VLSPLPV----------QSQTDMLPDTVVMTPTQSQLQDQLQRKHDELQKLILQQQNELRIVSEQLLLSRYTYLQPMMSMGFAPG
```

```
 M.domestica  VQPV--IAPFQAAP-VISPLPH----------HPQHEMIAAPVVMTPSQNQLQEQLQRKHDELQKLILQQQDELRIVSEQLLLARYTLLQPMMPVNY--G
    A.pernyi  QHGIGAQYLEPSPYVGTVAVP------------GVLPLSLQSLPIIVSPDQAQLQLQRKHEELQQMIVRQQEELRQVKEQLLLARLGILQPIINVNPIQH
 D.plexippus  QHGIGAQYLEPAPYVGAVGVP------------AVLPLSLPPIPVIVAQDQAQLQLQRTHRELQQMIVRQQEELRQVKEQLLFARLGILQPVINV---QD
  R.prolixus  PQSVLNVLPP-QR--------------------------LDQ-------------LERRQEELQQRIVEQQTELRHVTEQLFMAKYGLFPPVLNTLPYTV
 T.castaneum  MQPVISGFSPIQP--------------------------LDNVMLTPAQTQMQIDLQRKHAELQAIIGQQQAELRRVSEQLLMARLGLLPQNQPVQNYSP

L.longipalpis  SVPSNRQSI-----NSTVTHLHATPAQNAVPMIEHPEQDGSMTTDQGGGEIVSYMQLNPVPSSHLQQQNQQQH---SDAEPLSYQMPDQGQIMFDPGNLR
   Ae.aegypti  S------S--------STIDISSRCSSSTSYLQHHQSSHSSHQYPTH------PSLANQPQNLLNPQP---------HQSQQ-----QPDQQMSI-PLDQK
   An.gambiae  T------GNGPGPGPSVGPPGERCASGGSFLQAHHQGNSYHHHHYH------PSAAQAQQQQQQQQQQHPPSHGGVHHAQQPQHPLQDQQQLCIGPPDQK
D.melanogaster  NMTAAAVGNLGASGQRGLNFTGSNAVQPQFNQYGFALNSEQMLNQQDQQMMMQQQQNLHTQHQHNLQQQHQSHSQLQQHTQQQHQQQQQQQQQQQQQQQQ
  M.domestica  QTDALTLGN----NSRNINFN-NNAVQQQFNQYGFAMNSNEMNNQQ---IMLQHQQNLQQQLQHNQAQRHQCQ---QQQQQQQHQASQQLQANMANTTNA
     A.pernyi  SYVNREEAQGNPRFPSLPEEYDRQIRDNNTYQQRRAPNPDRNNLP
  D.plexippus  PFTNPEQM---PNRSSIMYDGNRQLSYPQTSHQQNHNMPPQ
   R.prolixus  SQTVPSSEMYVAQAIPPVIIEPHQVRVDEEQPSEHYQVPHQSGILYNPPVNSPNSTTQTNH
  T.castaneum  QVSIAYQGSNSGTVSSTTTTIPGIQSSAVGSPSLIVPVSISLPIPQHQPNMLYTPSDSNGQTK

L.longipalpis  SPSSEPHSQ
   Ae.aegypti  PIFHPN-QQLNFTDTGQPKLPGDPNSEAISYMQ-----LTPVPIHHLQQQSQPLQQQQQHHHQMQNPGMNLPGPSGNSMELLQYQ--------------M
   An.gambiae  PILHPDPEQLNFTDTGQGKL-GEPN-EMISYMQ-----LTPVPLHHLQHQSSQSSTSSTPATSMMSQQQKQHQQQQHQQQQQHMQ--------------M
D.melanogaster  QQQQQQQQQQQQQQQQQLQLQQQNDILLREDIDDIDAFLNLSPLHSLG-SQSTINPFNSSSNN-NNQSYNGGSNLNNGNQNNNNR--------------S
  M.domestica  ASNHPPHQPIHSEEPDEMLLNRNVPDLEQFAQEEFDAFLNLSPLQNIGTSEPTLNSISATVQNPNSANPNTPANITSTPNPLNSAGSGHIMETTPYKGDS
     A.pernyi
  D.plexippus
   R.prolixus
  T.castaneum

L.longipalpis
   Ae.aegypti  AEEQAQTLFTSGMEQQQQQQQHLPQPQQQHHHHQQQQQQQMHHQHSH--------------HHQHHHQHHHHQQQQQQQQQQPQHHQSQHHHSQSSSVGS
   An.gambiae  GAPPDGGTGAGGNSNNNSNGNTIGLLQYQM---AAEQAQILFTSGME--------------QQQGQQQQQPAVQQQQQQQQQQQHQGAGSSDAGSRTCPS
D.melanogaster  SNPPQNNNEDSLLSCMQMATESSPSINFHMGISDDGSETQSEDNKMM--------------HTSGSNLVQQQQQQQQQQQILQQHQQQSNSFFSSNPFLN
  M.domestica  NNAAATTSEDSIFSYIQLAAESSHPMNFNMNISDDITDANAEDNKLMSHSHSANNERSNINHADHHQLALPNPQPTSQQNTSPLGPQQSQRSTLFAACQS
     A.pernyi
  D.plexippus
   R.prolixus
  T.castaneum

L.longipalpis
   Ae.aegypti  PGGSTRSHPRSDM
   An.gambiae  QTSEM
D.melanogaster  SQNQNQNQLPNDLEILPY-QMSQEQSQNLFNSPHTAPGSSQ
  M.domestica  SPRTNDVQLPNTTAHQPTSQIGNNSGSNNNNNPTSNNNDSSILSNTMNRILPFQIASEPLENIFNTANSSTQQSGGTNP
     A.pernyi
  D.plexippus
   R.prolixus
  T.castaneum
```
