# Supplementary material for: Clocks do not tick in unison: isolation of Clock and vrille shed new light on the clockwork model of the sand fly Lutzomyia longipalpis
Source: Parasit Vectors. 2015 Oct 6;8:505. doi: 10.1186/s13071-015-1117-6 (PMC4595053; doi:10.1186/s13071-015-1117-6)
Supplement: Additional file 4: — Additional file 2. Multiple sequence alignment of VRI orthologues in L. longipalpis [GenBank: KR706372], Ae. aegypti [GenBank:XP_001661622], An. gambiae [GenBank:XP_317705], D. melanogaster [GenBank:NP_477191], M. domestica [GenBank:XP_005180212], R. prolixus [VectorBase:RPRC000393], D. plexippus [GenBank:AAT86041], A. pernyi [GenBank:AAS92609] and T. castaneum [GenBank:EFA11543]. The Highlighted region (blue) corresponds to bZIP functional domain. Text colors correlate to conservation thresholds for a position. In red font, highly conserved residues (threshold=90 %). In blue font, weakly conserved residues (threshold=50 %). (PDF 171 kb) [file 13071_2015_1117_MOESM4_ESM.pdf]

```
L.longipalpis
Ae.aegypti
An.gambiae
D.melanogaster  MSIVCTLEQKVNFFKAAATTKNLLILKNN---------------TDTNYINNYKQDN--------PSNNKFPRIQAQSNNSHLQHQQQLQQKLAQLHHYS
M.domestica     MSIVCTLEQKVNLFKGPVATKNLLILKNNINNNPQITPAATTTTTTANSTTNSTNNNYSSAVTTLPSGKLLTDIKSSSSSSGAPISVSSLKEKSLLSSAP
A.pernyi
D.plexippus
R.prolixus
T.castaneum

L.longipalpis   MHPDPGIIFSASPGVLRQRMMVAGFVERQQSCS
Ae.aegypti      MMVADFVSRPQNGS
An.gambiae      MMVADFVTRQQNGS
D.melanogaster  QQKLSGSDFP----YGPRPPTGGKEEKLLLLAPPGKLYPEASVSTAMPEVLSGTPTNSHNKANIAMMNN--------------VRLSNISPTLSMNGSSN
M.domestica     NHLIKGQDFKNRLTVTPTTELLQLHQQQQQQQQQHQQHLLLTAAANMPEVLQAAAANSLTSNSTKYHSNPAQGLVTPATAAAMVRMSTMSPTLSMNGSSN
A.pernyi        MVAEFILSQQQLL
D.plexippus     MVAEFILSQQQLL
R.prolixus      MVAEYMER-QSSSPLNGETPVLNGSTP
T.castaneum     MVAEFVGRGQSGSPVNGEIPQLNSNMP

L.longipalpis   PSNGEGPPMGHHQMHSPRGHMEMYHEHTSSMSPRSPGADSYGQREPGGNQNYEIGSAAHLKRKELFTQRKQREFIPDNKKDESYWDRRRRNNEAAKRSRE
Ae.aegypti      PINGEIPGQNHN-NSNMPAHSALLMSNNST-----GRASP-ASPESDYNGN-GYDLSGHLKRKDLFSQRKQREFIPDNKKDDSYWDRRRRNNEAAKRSRE
An.gambiae      PLNGEIPGQNHNSNSNMPAHSALLMSNNSSNSNSSGRASPHTSSESEFPQN-SYELAAHLKRKELFSQRKQREFIPDNKKDDSYWDRRRRNNEAAKRSRE
D.melanogaster  EASNLHPLSMYGGSISPQSNDSGMSDSLGKYVPGSGYGDGMMAQSPSQGGN-GPQSALTAAQKELFSQRKQREFTPDNKKDESYWDRRRRNNEAAKRSRE
M.domestica     EATNVHSLSMYNGPVSPQSSDSGHSMSDMQHNNYMNYNPDFTGMTKQQ----------VSAAKELFSQRKQREFTPDCKKDDSYWDRRRRNNEAAKRSRE
A.pernyi        SAGGGAALGPAPVQTRAPPPPRARLSVSPHFPMDQSHPNNGSPENPEDY-SSFDF-K----RKEFFGQRKQREFIPDNKKDDGYWDRRRRNNEAAKRSRE
D.plexippus     NAGGGPPLVPPPSPSRIAQPPRACLSVSPHFPMEQGPPSS----NGDDYGNSFDFSK----RKDFFGQRKQREFIPDSKKDDGYWDRRRRNNEAAKRSRE
R.prolixus      SSSNTGSMALPTGYYVIDGPGSVEMSSNGPAATTTASAEHDGVQGTGEYCQNGSYEMNSLARKDLFSHRKQREFIPDNKKDESYWDRRRRNNEAAKRSRE
T.castaneum     ASHGRELLSSSTSPDADSFPPGYNMNSVKH--------------------------------KELFSQRKQREFTPDSKKDDSYWDRRRRNNEAAKRSRE

                       bZIP
L.longipalpis   KRRFNDMVLEQRVVELTKENHVLKAQLDAIKDKYNICGESLVSVDQIMATLPNADQLLTITKRAKL---------------------------------
Ae.aegypti      KRRFNDMVLEQRVVELTKENHVLKAQLDAIKNKYNICGENLVSVDQIMATLPTNEQVLSSTKRVKI-NSTPSIVFPLARPTVPQSPLTPQQGPQIPS-LH
An.gambiae      KRRFNDMVLEQRVVELTKENHVLKAQLDAIKSKYNICGENLVSVDQIMATLPTNEQVLSSTKRVKLANGQARSSSPTGSMIGFPLSRSPSRQPQATSPSQ
D.melanogaster  KRRYNDMVLEQRVIELTKENHVLKAQLDAIRDKFNISGENLVSVEKILASLPTSEQVLSNTKRAKMSGSGGSSSGSSPSGSGSGEGSPQGGHNGYPVGPP
M.domestica     KRRYNDMVLEQRVVELTKENHVLKAQLDAIKDKFNISGENLVSVEQILASLPTSEQVLSTTKRSKLSTPATNFNGNANYGTSGNNLQTQRSSVDVLSSSH
A.pernyi        KRRFNDMVLEQRVVELSKENHVMKAQLDAIKEKYGICGENLISIDQVLATLPTCDQVLCVTKRSKLSSNA---LFSPAPPPPPPGPPASPPSPPPQRAPE
D.plexippus     KRRFNDMVLEQRVVELSKENHVLKAQLDAIKEKYGICGETLISIDQVLATLPTCDQVLCVTKRSKLSTNT---LFSPAQAPPAPVQPASPPSPQPQHAPE
R.prolixus      KRRFNDMILEQRVVELSKENHFLKAQLAAIKDKYGISGEAVVNVEQVMATLPTNDQVLSITKRAKINNNISPPIIFQPNSVPIPTPVIHKANPSPPLALN
T.castaneum     KRRFNDMVLEQRVVELTKENAILKAQLEAIKEEYGICGENVVCVEKVLANLPTNEQVLSLTKRQKLTHSANPLLFNPH---------------------

L.longipalpis   --------MAYPNSPSRQPPPPESPPPELHAPPLFAHRH---HTPPL---------------------------------HPALLQNPPGGDHSHDLHH
Ae.aegypti      SHQP----LSQPNVIQPSQQHPTQQPQQQSQQAAVIHPNPNIESPPPPQSIASQLQQI--TIQHTSSHYSHHSPA-AIYREY-EPLTNGSSRCDSNKHES
An.gambiae      SHGR----ASSPHSPPAANGTPHLQQTLLAVQAPVIHPNPNVESPPPAAAGSSPTVSSGGAIQHTNGGPAYLSPVHAIYHRN-STIIEYERASEGGKLTS
D.melanogaster  LSPL----IYGPNGNARPEATVKSVHHIHHAGVAPPPTHLQQLVVPQSQTQHLYQPQPQQHQPHQQQQISQP-PQQQQQQQEPSPSAGSSSPVISDPHNR
M.domestica     NDKLTAQGVIKPNG-AVAAPSVNGVSPLHHLPSAINAAAAAGHQPSIQATQALYVPASAASAVYVPANINESLSSNTPPHQHHSVVAAAATTTTSLVPTQ
A.pernyi        PYQER---LPVPEPYYPHPAHYEPPGSVLNLSRTRRAP-SPYELSSLSGSGDETAEPYAPENNCLPLKLRHKSHLGDKDVAS-ALLSLQHIKQEPGPRSS
D.plexippus     QYQER---LPAPEAYYSHPSHFEPPGSVLNLSRPRRAP-SPYELSSLSGSGDETTEQYAPENNCLPLKLRHKSHLGDKDVAS-ALLSLQHIKQEPGPRSS
R.prolixus      MYPED---QYSEERY---PFPFPPVLPPLNPPVLEAASNSVLNLSRRANSNVNVGVTTPVDMVSLPHKLRHKSHLGDKDVAATALLSLHNIKQEA--RAS
T.castaneum     -------------------IHYDSNDALNLSARSRSRGQSPFEVSSNSGDESAPVALTSEANNSLPLKLRHKSHLGDKDAAN-TLLSLQNIKQEPGPRAS

L.longipalpis   PSNPPMH-------------------------------------------------VYAPAPSYVHHQPTPAPHHPLPPAMSPEDPHHHTT--------
Ae.aegypti      TIRSPLTRVELMEHDEHRSEQLAEHRDRERERDRERNLDRNDVPVLPVP--------TFGSSNSHISGPHISHHPYSSPYTIPNYPVSYAAN-------L
An.gambiae      PAGSPPAHVPVAHAHSHHYSQHHHHHPH--------HLAAHHVHPPHFV--------VGASSPYASSALPASGYPLTAAAAAVAAYANGASA-------L
D.melanogaster  PPSTTIANLQVQLQQALNRNVRPEDLDSLRKVVAAGALYNAAAVVGAPP--------PPPSAGLYVPAPSAYKDHLEAAAAWS---HNVEAA-------V
M.domestica     TPTPNLQNLHVL--QALNRNC---DFENIRKVVAAAAVASNAAAADLPSTVSGLYVNPQGAAAAAIYAGATQHVHNPLYTKLSKEEHSYLSATMVEGPIV
A.pernyi        PSWDGEGSSDERDSGISLGVEY-------------RP--QPERIPEE-----EDAH-LKAELARLATEVATLKNMMNQNKARGHEH
D.plexippus     PSWDGEGSSDERDSGISLGVEY-------------RPPRSDERLAEE-----EDAH-LKAELARLATEVATLKNMMHQNKSRSLEH
R.prolixus      PPWDTEGSSDERDSGISLGAEW----IMPATTLPEQPSTSPLEEPIETEDNLDDTH-LKSEVARLASEVATLKSMLTRKKSAAININ
T.castaneum     PPWDAEGSSDERDSGISLGAEWSTQAAQAAATLQTLKQSHLQAMQEAAGGDAETKHRIHSEIVRLSSEVEHLKSMMIGKEKEPRH

L.longipalpis   ----------------REDALNLSRRPASP--FD---------------ASSGSCASGTASGDDEHCCESSTVV------------APPVVDIANSLPLK
Ae.aegypti      YPSPPREI---NAL-LTANVLNLSRRAPSP--YETSNGTG---------SNSSHS----SSGDDEHDREH-M------------------HDHNNSLPVK
An.gambiae      YGSPPRTTEIASAL-LTANVLNLSRRAPSP--YDASPAQT---------ASSTASAGSTSGGEEEPDREREI------------------HEHANSLPVK
D.melanogaster  SSSAVDAVSSSSVSGSAASVLNLSRRACSPS-YEHMLSSTT------SSTLSSASSSGAVSGDDEQEHEPAHMAPLQ----LQRSSPQQGSDANNCLPLK
M.domestica     SSSAGDSVSSSSPR-ETASVLNLSRRASSPTAYEHMLSSTTPTSSRLSSAMSSASSSGAVSGDDDHEADNEMAEETNEHNLASTTTSPHSNDSNNCLPLK
A.pernyi
D.plexippus
R.prolixus
T.castaneum
```

```
L.longipalpis   LRHKSHLGDKDAA-SALLALQHIKQEP-SLRASPP-WGDGEG--SSDERDSGISLSGGHGEPWVRKMPPGT-----------------------------
  Ae.aegypti    LRHKSHLGDKDAA-TALLALQNIKQEP-QIRSSSP-WDDGDG-GSSDERDSGIST-EWPTKAEQKMMVPLPVSPPSTSVSPIAS-------STANIIGG-
  An.gambiae    LRHKSHLGDKDAA-TALLALQNIKQEPIGHRSSSPAWDDGDG--SSDERDSGISTNEWPTKAEQKMMVPLPGSPPSAASSSSSAASGGAIVSTAAMVAAV
D.melanogaster  LRHKSHLGDKDAAATALLSLQHIKQEPNCSRASPPAWNDG-GDNSSDERDSGISIASAEWTAQLQR----------------------------------
 M.domestica    LRHKSHLGDKDAAATALLALQHIKQEPISNRASPPAWADNSGDNSSDERDSGISIPSPEWAAQFQR----------------------------------
    A.pernyi
 D.plexippus
  R.prolixus
 T.castaneum

L.longipalpis   -TTTLAVGVVGILDKEEENCHLKTQLKRLENEVASIKNMMILSNAN---GATAAAQ
  Ae.aegypti    -KITSIPASVVISKKAEENIHLQSKLARLESEVATIKNMMISNTAGSGFGVTAAAQ
  An.gambiae    AKITSIPASVVISKKAEENIHLQSKLARLESEVATIKNMMISNTTGSGFGVTAAAQ
D.melanogaster  -KLLAPKEANV-VTSAERDQMLKSQLERLESEVASIKMILAE
 M.domestica    -KDTCTATAVVTVTPSEREHILKSKLARLESEVATIKNDLYSRSKCCT
    A.pernyi
 D.plexippus
  R.prolixus
 T.castaneum
```
